# The Elixir of Life

**Published:** 1889-09

**Authors:** 


### THE ELIXIR OF LIFE.

It is one of the proud boasts of the present day that we live in an age of reason, an age unfavorable to fables, superstitions and chimerical fancies. But there never was a greater mistake. Men now indulge in as absurd notions, as fanciful theories and as ridiculous modes of thought as ever they did in the palmiest days of chivalry. The search for the philosopher's stone has ceased only to give place to the ridiculous fads of modern therapeutics, while the dream of Ponce de Leon surpasses only in romantic beauty the dream of a modern French dotard.

The craze which has followed Brown-Sequard's announcement of his discovery of the elixir of life is based upon the inherent desire in the human breast for the prolongation of life and the rejuvenescence of old age, and also upon the *prestige* of a great name. In the eagerness of their instinctive desires men forget that old age is the fruitage of maturity as inevitably as manhood is the fruitage of youth, and that it would be as rational to attempt to prolong the duration of intra-uterine life or to postpone puberty by artificial means as to ward off senility by human endeavor. The impossibility of the one is but a corollary of the absurdity of the other.

Our condemnation of Brown-Sequard's theory is not based upon the repeated failures which have followed its application. To such failures we attach no significance. Scientific experimentation requires an accuracy of method and a precision of detail which are only to be gained by long experience. When undertaken by novices its results are valueless. Without a thorough, practical knowledge of the *technique* of microscopy and bacteriology, the determination of the existence of bacilli in the sputa of a tuberculous patient would be but mere guess work. The same is true of the practical solution of the question under discussion. Therefore such failures count for nothing for or against, except in the hands of experienced scientists who are capable of eliminating all sources of error. We have seen no reports of experiments upon this subject at the hands of such men. The absurdity of the theory is inherent in the theory itself and to the scientific mind is axiomatic. It must therefore be relegated to the domain of pseudo-science and thus be placed on a par with spiritualism, mind-reading, mesmerism and kindred delusions which engage the attention of *dilettante* scientists.

The sum total of human life is undoubtedly capable of increase through the instrumentality of the human testicle, but in the aggregate only and not in the individual. Meanwhile it remains as true to-day as in the time of David that "the days of our years are three score years and ten, and if by reason of strength they be four score years, yet is their strength labor and sorrow, for it is soon cut off and we fly away."

